# Supplementary material for: A physiological approach for assessing human survivability and liveability to heat in a changing climate
Source: Nat Commun. 2023 Nov 29;14:7653. doi: 10.1038/s41467-023-43121-5 (PMC10687011; doi:10.1038/s41467-023-43121-5)
Supplement: Supplementary file 1 — Supplementary Information [file 41467_2023_43121_MOESM1_ESM.pdf]

Supplementary Information

A physiological approach for assessing human survivability and liveability to heat in a changing climate

Jennifer Vanos,*[1] Gisel Guzman-Echavarria,[2] Jane W. Baldwin,[3,4] Coen Bongers,[5,6] Kristie L. Ebi,[7] Ollie Jay[6]

[1]School of Sustainability, Arizona State University, Tempe, AZ, USA
[2]School of Geographical Sciences and Urban Planning, Arizona State University, Tempe, AZ, USA
[3]Department of Earth System Science, University of California Irvine, Irvine, CA, USA
[4]Lamont-Doherty Earth Observatory, Palisades, NY, USA
[5]Department of Medical Sciences, Radboud university medical center, Nijmegen, The Netherlands
[6]Heat and Health Research Incubator, University of Sydney, Sydney, Australia
[7]Center for Health and the Global Environment, University of Washington, Seattle, WA, USA

***Corresponding Author**: Jennifer Vanos, jvanos@asu.edu*

This file includes:

1. Supplementary Note
2. Supplementary Methods
3. Supplementary Table
4. Supplementary Figures

1.0 Supplementary Note

The model code was developed using Python 3.10.9, and the authors thank the teams behind this open-source project, as well as NumPy, Matplotlib, Xarray, Pandas, and MetPy[1] developers. Custom codes with the model developed for this study and tutorials to reproduce survivability and liveability temperature-humidity matrices are available via Zenodo data repository (https://doi.org/10.5281/zenodo.10020136). The CMIP6 data were downloaded from https://esgf-node.llnl.gov/search/cmip6/. The Zenodo repository also contains the source data (also provided with this paper) used to create all the graphs in the main document and this supplemental material, as well as the input data for the personal profiles custom-built individual characteristics (which describes the anthropometrics, activities, clothing, sweat features, and acclimatization). The T_w values were estimated using the Davies-Jones method.[2]

2.0 Supplementary Methods

Model Details of Theoretical Model Approach & Fundamentals for Survivability & Liveability Analyses

Our approach leverages methods of partitional calorimetry to model human heat balance[3,4] describing heat transfer between the human body and the surrounding environment. Internal body heat storage (S) is

defined by the imbalance between internal metabolic heat production (H_{prod}) and the net heat loss from the skin surface to the surrounding environment (H_{loss}):

$$S = H_{prod} - H_{loss} \quad \text{(W)} \quad (1)$$

Also written as:

$$S = M - W_k - R - C - K - E \quad \text{(W)} \quad (2)$$

where H_{prod} is represented by $(M - W_k)$, or the difference between metabolic rate (M) and mechanical work (W_k). In assuming mechanical work (*i.e.*, energy transferred to a force) is negligible when sedentary (W_k= 0 W), then $M = H_{prod}$, and the sum of the remaining variables—all avenues of heat exchange at the skin surface—represent H_{loss}[5,6]. R, C, and K represent rates of dry heat loss by radiation, convection, and conduction, respectively. By convention, these values are input into equations (2) and (3) as losses; if they switch to heat gains, they will be positive values to add to the overall sum. E represents the rate of evaporative heat loss. K is assumed negligible as the proportion of skin surface area in direct contact with a solid surface is typically very small under most everyday activities. Hence, equation (2) becomes:

$$S = M - R - C - E \quad \text{(W)} \quad (3)$$

Heat transfer via radiation only occurs by skin (R_{skin}), whereas heat transfer via convection and evaporation occurs from both the skin (C_{skin}, E_{skin}) and the respiratory tract (C_{res}, E_{res}). Thus, equation (3) becomes:

$$S = H_{prod} - R_{skin} - C_{skin} - E_{skin} - C_{res} - E_{res} \quad \text{(W)} \quad (4)$$

where C_{res} and E_{res} are estimated by ASHRAE[7]:

$$C_{res} = 0.0014 \cdot M(34 - T_a) \cdot A_d \quad \text{(W)} \quad (5)$$

$$E_{res} = 0.0173 \cdot M(5.87 - P_a) \cdot A_d \quad \text{(W)} \quad (6)$$

where T_a is air temperature (°C), and A_d is the body surface area (m^2) estimated based on total body mass, m (kg), and height, H (m)[8]:

$$A_d = 0.202\, m^{0.425}\, H^{0.725} \quad (m^2) \quad (7)$$

where P_a is the water vapor pressure of the inspired air in kPa. A_d was 1.60m^2 for a young, healthy female adult on average, and 1.78m^2 for an older female adult. These surface areas arrive from equation 7 and are based on global average height for women[9] (~1.62m) and average weight data based on ExposFacts[10,11] and the EPA Exposure Handbook[12] (using NHANES data) to ensure a more representative global average, resulting in ~57kg for young female and ~74kg for older female.

The combined dry heat loss (R_{skin}+C_{skin}) is given by:

$$R_{skin} + C_{skin} = \frac{(T_{sk} - t_0)}{\left(R_{cl} + \frac{1}{(h_{c+r}) + f_{cl}}\right)} \mathrm{A}_d \qquad \text{(W)} \qquad (8)$$

where T_{sk} is mean skin temperature (°C), which in our model is set to 35°C assuming maximally vasodilated skin[6,13]; this assumption aligns with Sherwood and Huber[14]. Because of this assumption in the current form of the model, results are only applicable for exposures to warm and hot weather. Adapting the mean T_{sk} would be needed to make it applicable to cooler environments. R_{cl} is the dry heat transfer resistance of clothing (m^2°CW^{-1}); t_0 is the operative temperature (°C) (equation 9); h_{c+r} is the combined convective heat transfer coefficient (Wm^2°C^{-1}) or the sum of the convective and radiative heat transfer coefficients, h_c (equation 11) and h_r (equation 10), and f_{cl} is the clothing area factor if clothed (equation 13).

T_0 is estimated as[15,16]:

$$t_0 = \frac{(h_r T_r + h_c T_a)}{h_{c+r}} \qquad \text{(°C)} \qquad (9)$$

where T_r is the mean radiant temperature (°C).

h_r is given by:

$$h_r = 4\varepsilon\sigma \cdot A_{eff} \left[\frac{(T_{sk} + T_r)}{2} + 273.2\right]^3 \qquad (10)$$

where ε is emissivity (assumed to be 0.97 for humans), σ is Boltzmann's Constant (5.67 ×10^{-8} Wm^2 K^{-4}), and A_{eff} is the effective exposed area of the body, which is 0.70 for sitting (survivability assessment) and 0.73 for standing (liveability). A_{eff} is essentially a "view factor" referring to the fraction of body area exposed that can be used for heat transfer with the surrounding environment.

h_c is calculated based on wind velocity (v_{air}) as[16]:

$$h_c = 3.1 \text{ for } v_{air} < 0.2\ \frac{m}{s} \qquad (\mathrm{Wm^2 °C^{-1}}) \qquad (11)$$

for individuals sitting and not moving. Otherwise, if a person is moving, as in the liveability analysis, h_c is calculated as[17]:

$$h_c = 8.3 {v_{air}}^{0.6} \quad \text{for } 0.2 < v_{air} < 4.0\ \frac{m}{s} \qquad (\mathrm{Wm^2 °C^{-1}}) \quad \text{(light clothing)} \qquad (12)$$

where we maintain airflow at 1ms^{-1}.

Finally, we estimate the clothing area factor, f_{cl}, on the body (ratio of clothed body surface to nude)[18], as:

$$f_{cl} = 1 + 0.31\, I_{cl} \qquad \text{(ND)} \qquad (13)$$

where I_{cl} is the insulation value in clo, assumed to be 0.36 clo (light shorts and cotton T-shirt) for the liveability assessment.

Evaporative Heat Loss and Restrictions to E_{max}

Equation (2) can be rearranged to determine the amount of evaporative heat loss required for heat balance, or required evaporation (E_{req}), expressed as[3,6]:

$$E_{req} = M - R_{skin} - C_{skin} - C_{res} - E_{res} \qquad \text{(W)} \qquad (14)$$

However, E_{req} cannot always be achieved. Evaporative restrictions can exist due three factors (see Fig.S1).

1) high environmental humidity or biophysical evaporative heat loss limit ($E_{max_{env}}$).
2) the physiological capacity to saturate the skin surface in high humidity environments due to a limited maximum skin wettedness (ω_{max}).
3) the maximum rate at which sweat can be produced (S_{max}) in low humidity environments.

For the first evaporation restriction, a limit to evaporative heat loss is present due to the prevailing environmental conditions (*i.e.*, if, in humid environments, the skin-to-air water vapor pressure gradient is insufficient to enable E_{req} to be attained), even with maximal sweating. Thus, $E_{max_{env}}$ represents the maximum evaporative heat loss for a given thermal environment and clothing (equation (16)). In general, if $E_{req} \leq E_{max_{env}}$ (W), heat balance (*i.e.*, S=0) is possible. The fraction of E_{req} relative to $E_{max_{env}}$ can be expressed as the biophysical skin wettedness required (ω_{req}) for heat balance:

$$\omega_{req} = \frac{E_{req}}{E_{\max_{env}}} \qquad \text{(ND)} \qquad (15)$$

where $E_{max_{env}}$ is calculated as[3]:

$$E_{max_{env}} = \frac{(P_{sk,sat} - P_a)}{R_{e,cl} + \frac{1}{h_e \cdot f_{cl}}} A_d \qquad \text{(W)} \qquad (16)$$

where $P_{sk_{sat}}$ is the water vapor pressure at the skin surface when saturated with sweat (kPa), P_a is the partial pressure of water vapor in the air (kPa), $R_{e,cl}$ is the evaporative resistance of clothing (m^2kPaW^{-1}), and h_e is the evaporative heat transfer coefficient ($Wm^{-2}kPa^{-1}$).

The $P_{sk_{sat}}$ and P_a are calculated based on either T_{sk} or T_{air}, respectively. Hence, the given vapor pressure value, P, is determined with the respective temperature value, T, determined from an approximation to the Clausius-Clapeyron relationship, as follows:

$$P = \frac{e^{18.956-\left(\frac{4030.18}{T+235}\right)}}{10} \quad \text{(kPa)} \quad (17)$$

Because T_{sk} is constant at 35°C in our analysis, the $P_{sk_{sat}}$ is constant at 5.62 kPa.

h_e is estimated by:

$$h_e = h_c \cdot LR \quad (\text{Wm}^{-2}\text{kPa}^{-1}) \quad (18)$$

where LR is the Lewis Relation (16.5°CkPa^{-1})[19].

For the second type of evaporative restriction, the capacity to physiologically wet the skin and thus distribute sweat across the skin surface in humid environments (known as the maximum skin wettedness (ω_{max})) can be constrained by several factors. This E_{max} restriction is denoted here as $E_{max_{wet}}$, and alters $E_{max_{env}}$ according to physiological alterations in ω_{max} (either 0.85 for fully heat acclimatized or 0.65 for not heat-acclimatized or due to the effects of primary aging beyond 65 years[19,20]) to obtain $E_{max_{wet}}$:

$$E_{max_{wet}} = \omega_{max} \cdot E_{max_{env}} \quad \text{(W)} \quad (19)$$

Finally, for the third evaporation restriction, E_{max} in dry environments is constrained by the rate at which one can physiologically produce sweat per unit of time (S_{max}), which therefore constrains E_{max} at submaximal ω_{max} values and is denoted as $E_{max_{sweat}}$. For example, in very hot and dry environments, the sweating rate required to attain $E_{max_{env}}$ may not be physiologically possible, and E_{max} must be adjusted accordingly. S_{max} differs due to factors such as age, sex, and acclimatization status (Table 1). The following equation is used to estimate $E_{max_{sweat}}$ (modified from Morris et al.[20] and Foster et al.[21]):

$$E_{\text{max}_{sweat}} = \frac{S_{max} \cdot \lambda . \rho}{3.6}\, r \quad \text{(W)} \quad (20)$$

where S_{max} is 0.75L hr^{-1} (18–40 years) and 0.51 L hr^{-1} (65+ years)[22], λ is the latent heat of vaporization of sweat (2,426 J·g^{-1}), ρ is the density of sweat (assumed as equivalent to water density of 1 kg·L^{-1}), and *r* is the sweating efficiency, which is the proportion of sweat produced that evaporates from the skin surface (not dripping off), thus contributing to evaporative heat loss. Finally, 3.6 is a conversion factor to convert time from hours to seconds and the mass unit conversion from g to kg (i.e., 3600 s·h^{-1}, and 1000 g·kg^{-1}). Sweating efficiency is determined based on the biophysical ω_{req}[23] (equation (15)), where:

$$r = 1 - \left(\frac{\omega_{\text{req}}{}^2}{2}\right) \text{ when } \omega_{req} < 1, \text{otherwise } r = 0.5 \quad \text{(ND)} \quad (21)$$

r = 0.5 is the minimum sweating efficiency[24]. The sweating rate required (S_{req}) to support a given E_{req} is estimated as:

$$S_{req} = \frac{\frac{E_{req}}{r}}{\lambda} 3.6 \qquad \text{(L h}^{-1}\text{)} \qquad (22)$$

Conditions are survivable if $S_{req}<S_{max}$. Note that it is assumed that lost water is replenished sufficiently to avoid a level of dehydration that would aggravate thermal or cardiovascular strain (*i.e.,* less than 2% of total body mass)[25].

The main paper describes how this model is applied to determine survivability as a dichotomous variable (survivable or non-survivable) (see Fig.S1), which is then accompanied by an integer as a flag that provides the reasons based on the physiology constraints (survivability zones 1 to 5) (*e.g.,* Fig.2 in main manuscript).

Applying Restrictions to Evaporative Heat Loss in Liveability Analysis

E_{max} will either be constrained in high humidity environments by the physiological limit to ω_{max} ($E_{max_{wet}}$, equation 19) or in low humidity environments by the physiological limit of sweating (equation 20; $E_{max_{sweat}}$) (Fig.S1). Hence, the lower of $E_{max_{wet}}$ or $E_{max_{sweat}}$ is used as the $E_{max_{lim}}$ in the final M_{max} estimation, equation (23). Note that with the use of $E_{max_{lim}}$, we assume S=0 in the liveability analysis, thus:

If $E_{req} \leq E_{max_{lim}}$ or $\frac{E_{req}}{E_{max_{lim}}} \leq 1$ then:

$$M_{max} = E_{max_{lim}} - H_{loss} \qquad \text{(W)} \qquad (23)$$

where H_{loss} can be represented by (H_{dry} +C_{res} +E_{res}) or (E_{req} + H_{prod}). M_{max} is a continuous variable (W), which we convert to an energy expenditure in METs for a simpler interpretation. Further details of the liveability algorithm are shown in Fig.S1.

Considerations of New Model Estimating Physiological Survivability Limits and Liveability

Similar to the existing 35°C T_w model[14], our survivability model estimates the heat stress effects of a single time window of climatic exposure (*e.g.,* 3- or 6-h). Accordingly, there is no carryover of T_{core} elevations from one time window to the next, and it is assumed that a person is starting each time window in a normothermic state (a core temperature of 37˚C). Such an approach was used so that any effects of future elevations in heat stress would not be overestimated. Humans have a distinct ability to adapt to hot environments (heat acclimatization) through physiological adjustments such as increasing sweating capacity[26]. To capture this phenomenon, key parameters in both our survivability and liveability models were set to be representative of a fully heat-acclimatized person[15] for healthy, young female adults.

While assumptions must be used in this approach, our new physiological survivability limit model represents a significant improvement over the widely used T_w of 35°C by accounting for:

1. Physiological constraints to sweat production in hot, dry conditions.
2. Sweating restrictions due to aging, which are the most-wide scale thermoregulatory impairment in society.
3. Sun exposure.
4. Different morphology between different ages.
5. A physiologically plausible core temperature threshold for heat stroke
6. The ability to behaviorally adapt by reducing metabolic rate (in our liveability analysis)

The T_w of 35ºC threshold model does not account for these aspects.

This paper and others using limits to adaptability or survivability frameworks (Table 1 main paper) focus on heat stroke deaths (hyperthermia) and do not model the two other common types of heat-related deaths: death from cardiovascular collapse and renal failure and collapse. Briefly, cardiovascular collapse is common in heat-exposed people with underlying cardiovascular disease[27] and is not necessarily driven by critically high core temperatures but by making an already compromised heart to work harder[28]. Finally, a heat-related death from renal collapse and failure may also occur in people with pre-existing kidney disease that is not necessarily due to thermoregulatory insufficiency[29]. These types of deaths are not directly considered in this work (a limitation of the current model) but are worthy of consideration in future work.

3.0 Supplementary Table

Table S1: Sun-exposed physiological limit based on new wet-bulb survival temperature (T_w), as modeled in the present study, and differences from the 35°C T_w survivability assumption [i.e., (T_w–35)]. Differences are stratified by exposure duration (3 or 6 hours) and age group, depicting a stark underestimation of impacts using the traditional T_w of 35°C survivability model as conditions become drier and with aging.

			Sun-exposed/Outdoors					
		**Relative humidity (%)**	**10%**	**25%**	**50%**	**75%**	**90%**	**100%**
**Exposure Time**	**Age**							
**3-hour**	18–40 yr	**T_w limit (°C)**	23.5	28.7	31.9	33.0	33.3	33.4
		**ΔT_w (T_w–35) (°C)**	(–11.5)	(–6.3)	(–3.1)	(–2.0)	(–1.8)	(–1.6)
		**Corresponding T_{air} (°C)**	49.2	46.6	41.3	37	34.7	33.4
	>65 yr	**T_w limit (°C)**	19.9	25.4	30.4	32.4	32.8	33.0
		**ΔT_w (T_w–35) (°C)**	(–15.1)	(–9.6)	(–4.6)	(–2.6)	(–2.2)	(–2.0)
		**Corresponding T_{air} (°C)**	42.9	42.1	39.6	36.3	34.2	33
**6-hour**	18–40 yr	**T_w limit (°C)**	22.6	28.0	31.5	32.4	32.7	32.7
		**ΔT_w (T_w–35) (°C)**	(–12.4)	(-7.0)	(-3.5)	(-2.6)	(-2.3)	(-2.3)
		**Corresponding T_{air} (°C)**	47.7	45.6	40.9	36.3	34.1	32.7
	>65 yr	**T_w limit (°C)**	18.8	24.0	29.4	31.5	31.9	32.1
		**ΔT_w (T_w–35) (°C)**	(–16.2)	(–11.0)	(–5.6)	(–3.5)	(–3.1)	(–2.9)
		**Corresponding T_{air} (°C)**	40.8	40.2	38.4	35.4	33.3	32.1

4.0 Supplementary Figures

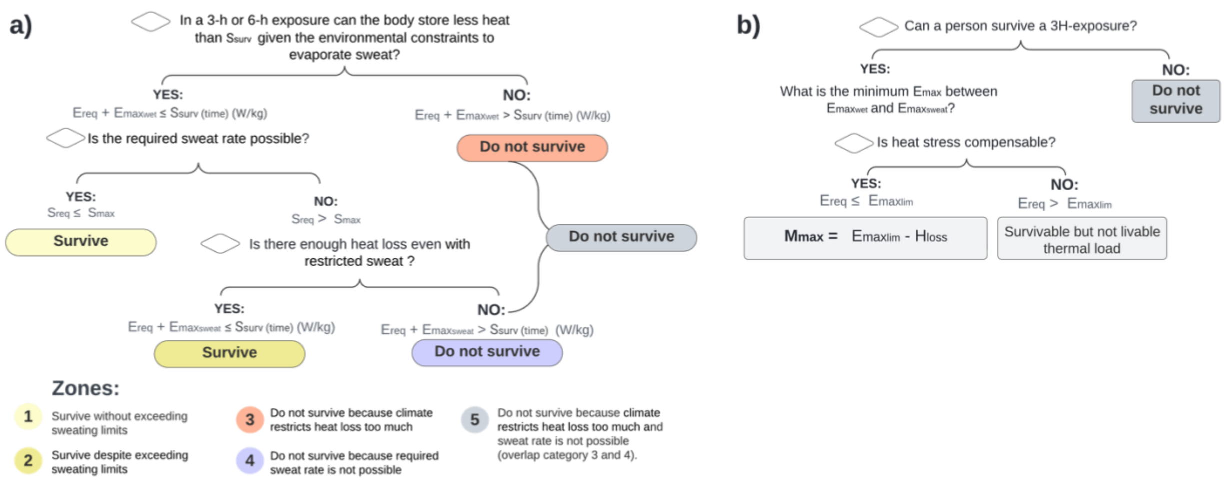

Figure S1: Model workflows for (a) survivability and (b) liveability. Survivability workflow shows how the model determines the conditions under which humans would survive based on limits to maximum evaporative heat loss (E_{max}) as connected to physiological constraints. Model workflow for liveability outlines how the model handles sweating restrictions and compensable conditions. $E_{max_{wet}}$ is the E_{max} restriction due to limited maximum skin wittedness; $E_{max_{sweat}}$ is the E_{max} due to sweating limitations. $E_{max_{lim}}$ is the lower of the two. E_{req} is required evaporation; H_{loss} is the net heat loss from the skin surface to the surrounding environment. Survivability output is a dichotomous variable (yes/no), accompanied by an integer as a flag that explains the result according to physiology constraints (categorical variable). Liveability output (M_{max}) is a continuous variable given in Watts or METs.

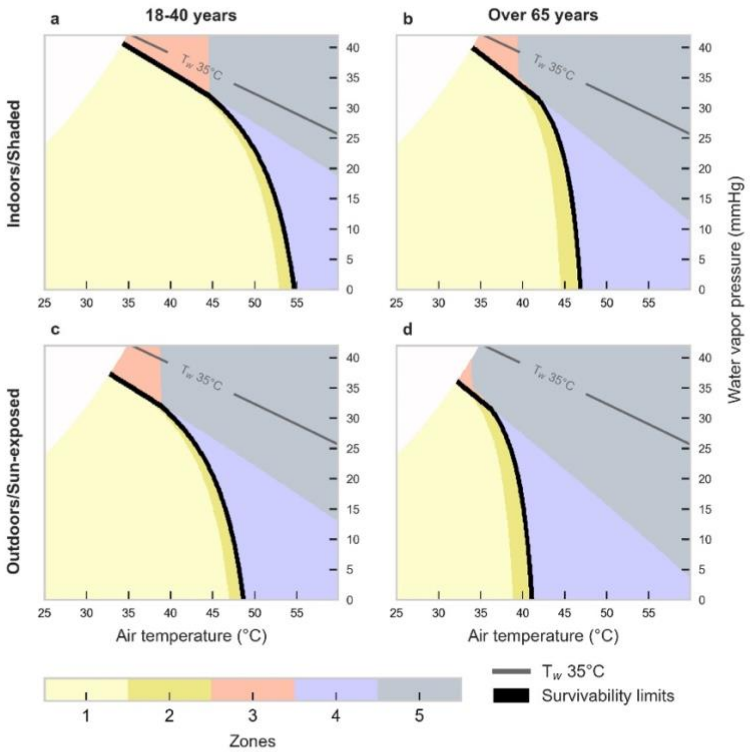

Figure S2: Same as Fig. 2 except with water vapor pressure as y-coordinate. New heat stroke survivability limits (thick black line) across 6 hours of constant exposure for young (a, c) and older female adults (b, d) in shaded/indoor (row 1) and sun-exposed/outdoor (row 2) conditions. Zones 1 and 2 represent areas of survivability, whereas zones 3–5 are non-survivable areas because of evaporative restrictions from the environment (zone 3), sweating limits (zone 4), or both (zone 5). T_w=35°C line is shown by thin gray line. White areas in top left indicate supersaturated conditions, which are unrealistic and are hence masked out. The new survivability limits illustrate the environmental conditions in which the body would reach a deadly T_{core} (i.e., after reaching uncompensable heat stress and accumulating enough heat to increase base T_{core} by 6°C, from 37°C (normothermia) to 43°C (heat stroke)). Source data for figures are provided with this paper.

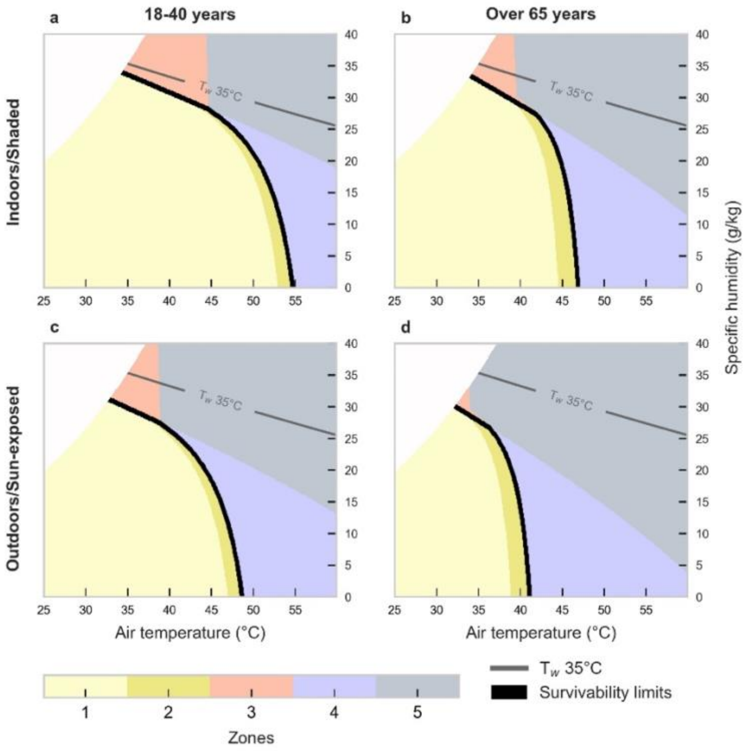

Figure S3: Same as Fig. 2 except with specific humidity as y-coordinate. New heat stroke survivability limits (thick black line) across 6 hours of constant exposure for young adults (a, c) and older female adults (b, d) in shaded/indoor (row 1) and sun-exposed/outdoor (row 2) conditions. Zones 1 and 2 represent areas of survivability, whereas zones 3–5 are non-survivable areas because of evaporative restrictions from the environment (zone 3), sweating limits (zone 4), or both (zone 5). T_w=35°C line is shown by thin gray line. White areas in top left indicate supersaturated conditions, which are unrealistic and are hence masked out. The new survivability limits illustrate the environmental conditions in which the body would reach a deadly T_{core} (i.e., after reaching uncompensable heat stress and accumulating enough heat to increase base T_{core} by 6°C, from 37°C (normothermia) to 43°C (heat stroke)). Source data for figures are provided with this paper.

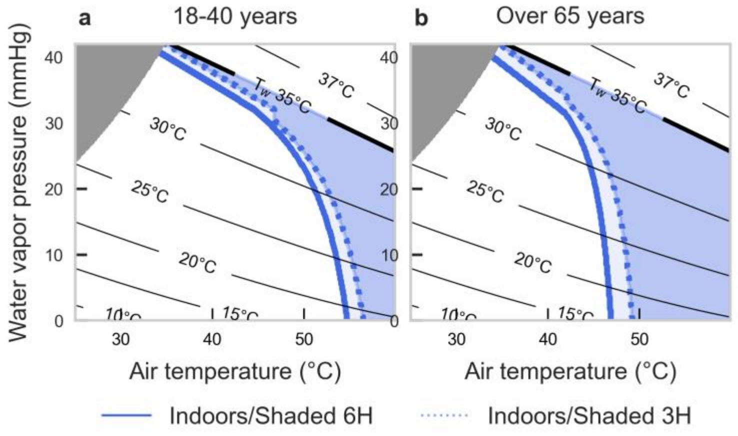

Figure S4: Same as Fig. 3 except with water vapor pressure as y-coordinate. Comparison of adaptability limit assumption (T_w= 35°C) and the new physiological survivability limits (blue-shaded area) for 3 hours (dashed lines) and 6 hours (solid lines) of constant exposure at given temperature and humidity combinations within shaded/indoors conditions. Graphs indicate model survivability for (a) young and (b) older female adult populations. Grey areas in top left indicate supersaturated conditions, which are unrealistic and are hence masked out. The new survivability limit is based on reaching uncompensable heat stress and accumulating enough body heat for a fixed time of constant exposure to increase T_{core} by 6°C (from 37°C (normothermia) to 43°C (heat stroke)). Black lines show T_w values up to 37°C to avoid unrealistic conditions (thick line shows T_w of 35°C). Source data for figures are provided with this paper.

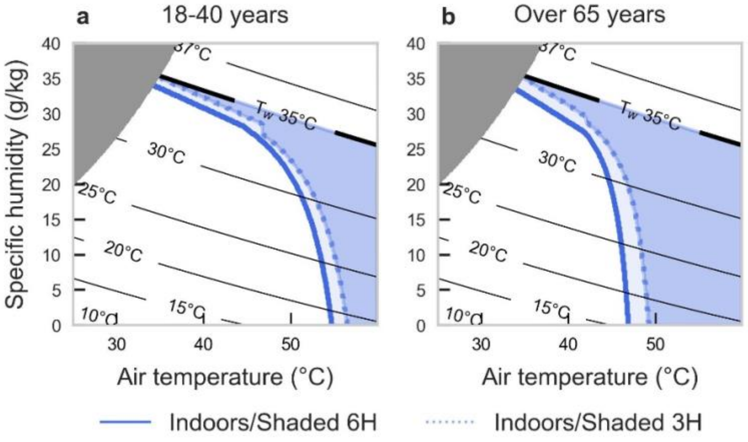

Figure S5: Same as Fig. 3 except with specific humidity as y-coordinate. Comparison of adaptability limit assumption (T_w= 35°C) and the new survivability limits (blue-shaded area) for 3 hours (dashed lines) and 6 hours (solid lines) of constant exposure at given temperature and humidity combinations within shaded/indoors conditions. Graphs indicate model survivability for (a) young and (b) older female adult populations. Grey areas in top left indicate supersaturated conditions, which are unrealistic and are hence masked out. The new survivability limit is based on reaching uncompensable heat stress and accumulating enough body heat for a fixed time of constant exposure to increase T_{core} by 6°C (from 37°C (normothermia) to 43°C (heat stroke)). Black lines show T_w values up to 37°C to avoid unrealistic conditions (thick line shows T_w of 35°C). Source data for figures are provided with this paper.

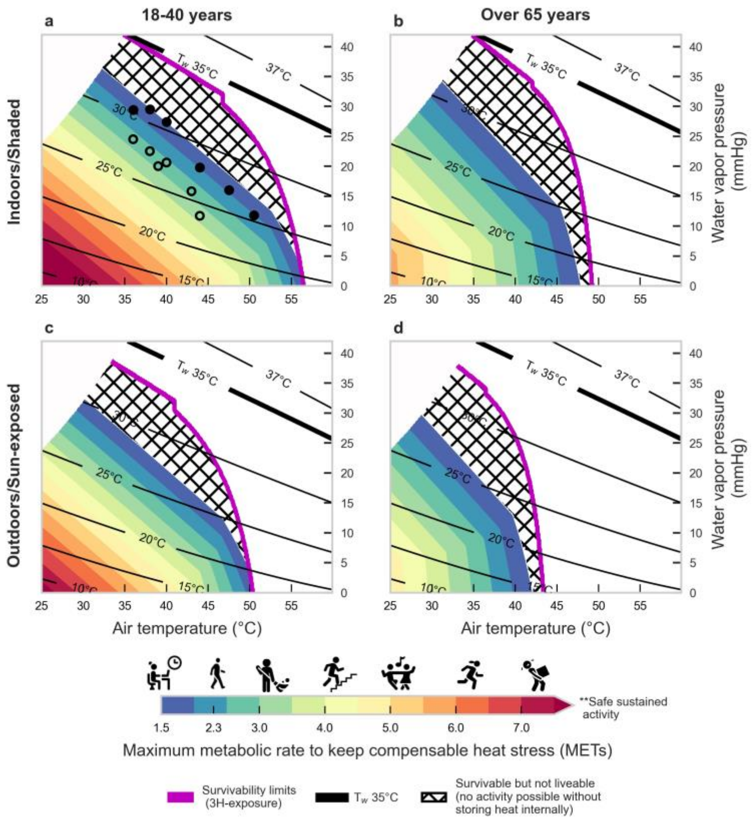

Figure S6: Same as Fig. 4 except with water vapor pressure as y-coordinate. Liveability estimates based on maximum safe metabolic rate (M_{max}, in MET equivalents (METs)) that a person can generate without a sustained rate of heat storage even with a maximal thermoregulatory response. Results are presented across a range of temperature and water vapor pressure (see Fig.4 for relative humidity) for younger (a, c) and older (b, d) female adults in shaded (top) or sun-exposed (bottom) steady-state environments. The 3-hour survivability line is shown in purple; constant T_w values are shown by the solid black lines until 37°C to avoid unrealistic conditions, with T_w=35°C highlighted by the thick black line. The hatched area indicates conditions that are survivable but not livable (*i.e.*, people cannot increase their activity without continuously storing heat, which may cause a rise in core temperature, but heat stroke death after a 3-hour exposure would not occur). Icons indicate MET-equivalent activities according to Ainsworth et al.[30] Circles indicate critical T_w limits reported by Wolf et al.[31] for minimal (~1.8 METs–filled circles) and light physical activity (~3.2 METs–open circles). Note: 1 MET corresponds to complete rest. Grey areas in top left indicate supersaturated conditions, which are unrealistic and are hence masked out. Icons provided by Icons8 (https://icons8.com). Source data for figures are provided with this paper.

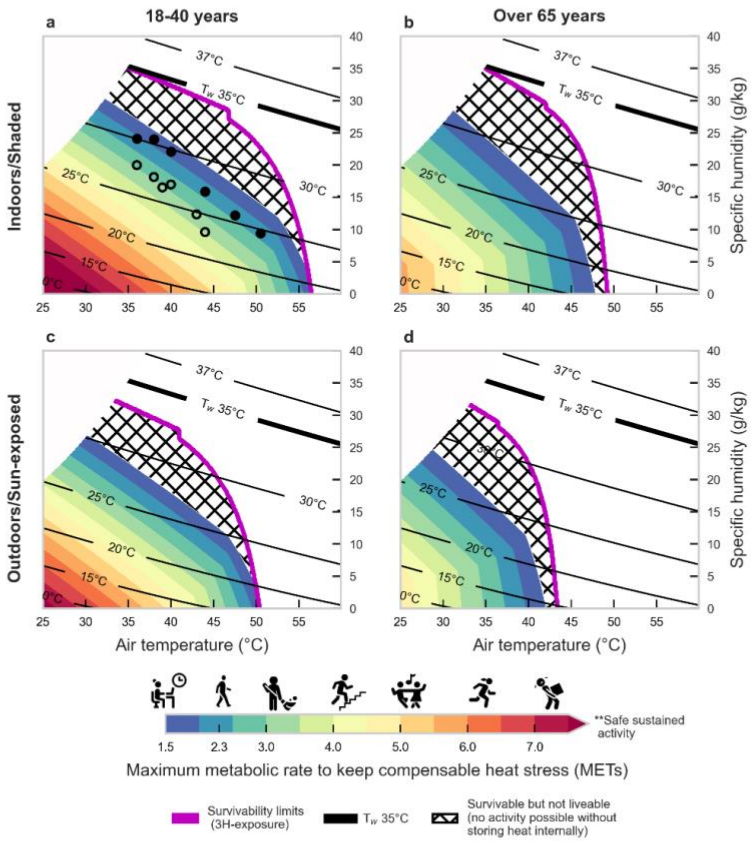

Figure S7: Same as Fig. 4 except with specific humidity as y-coordinate. Liveability estimates based on safe maximum metabolic rate (M_{max}, in MET equivalents (METs)) that a person can generate without a sustained rate of heat storage even with a maximal thermoregulatory response. Results are presented across a wide range of temperatures and specific humidity (see Fig.4 for relative humidity) for younger (a, c) and older (b, d) female adults in shaded (top) or sun-exposed (bottom) steady-state environments. Activities by MET level range from no activity (sitting ~1.5 METs), to housework (~3.0 METs), dancing (~5.0 METs), and heavy lifting (~7.0 METs). The 3-hour survivability line is shown in purple; constant T_w values are shown by the solid black lines, with T_w=35°C highlighted by the thick black line. The hatched area indicates conditions that are survivable but not livable (*i.e.*, people cannot increase their activity without continuously storing heat, which may cause a rise in core temperature, but heat stroke death after a 3-hour exposure would not occur)). Icons indicate MET-equivalent activities according to Ainsworth et al.[30] Circles indicate critical T_w limits reported by Wolf et al.[31] for minimal (~1.8 METs–filled circles) and light physical activity (~3.2 METs–open circles). Note: 1 MET corresponds to complete rest. [29][30]Grey areas in top left indicate supersaturated conditions, which are unrealistic and are hence masked out. Icons provided by Icons8 (https://icons8.com). Source data for figures are provided with this paper.

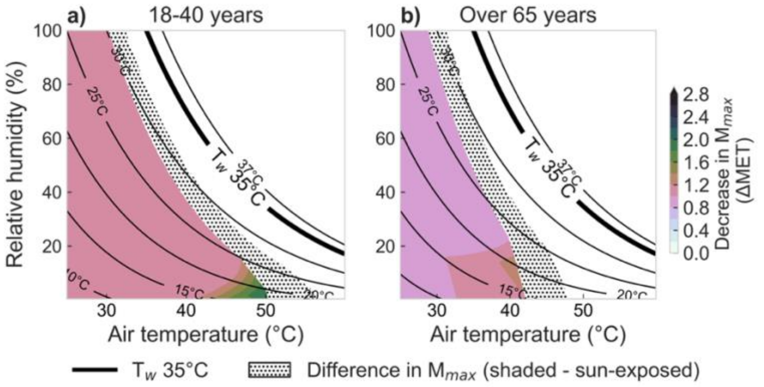

Figure S8: The difference in liveability (M_{max}) between sun-exposed or shaded conditions for young (a) and older (b) female adults. The dotted area indicates T_{air} and RH combinations where conditions are liveable (and survivable) in the shade, but a change to sun-exposed conditions shifts to only survivable (*i.e.*, they cannot perform any activity without continuously storing heat internally). Note that radiation was modeled assuming partly-cloudy conditions. Source data for figures are provided with this paper.

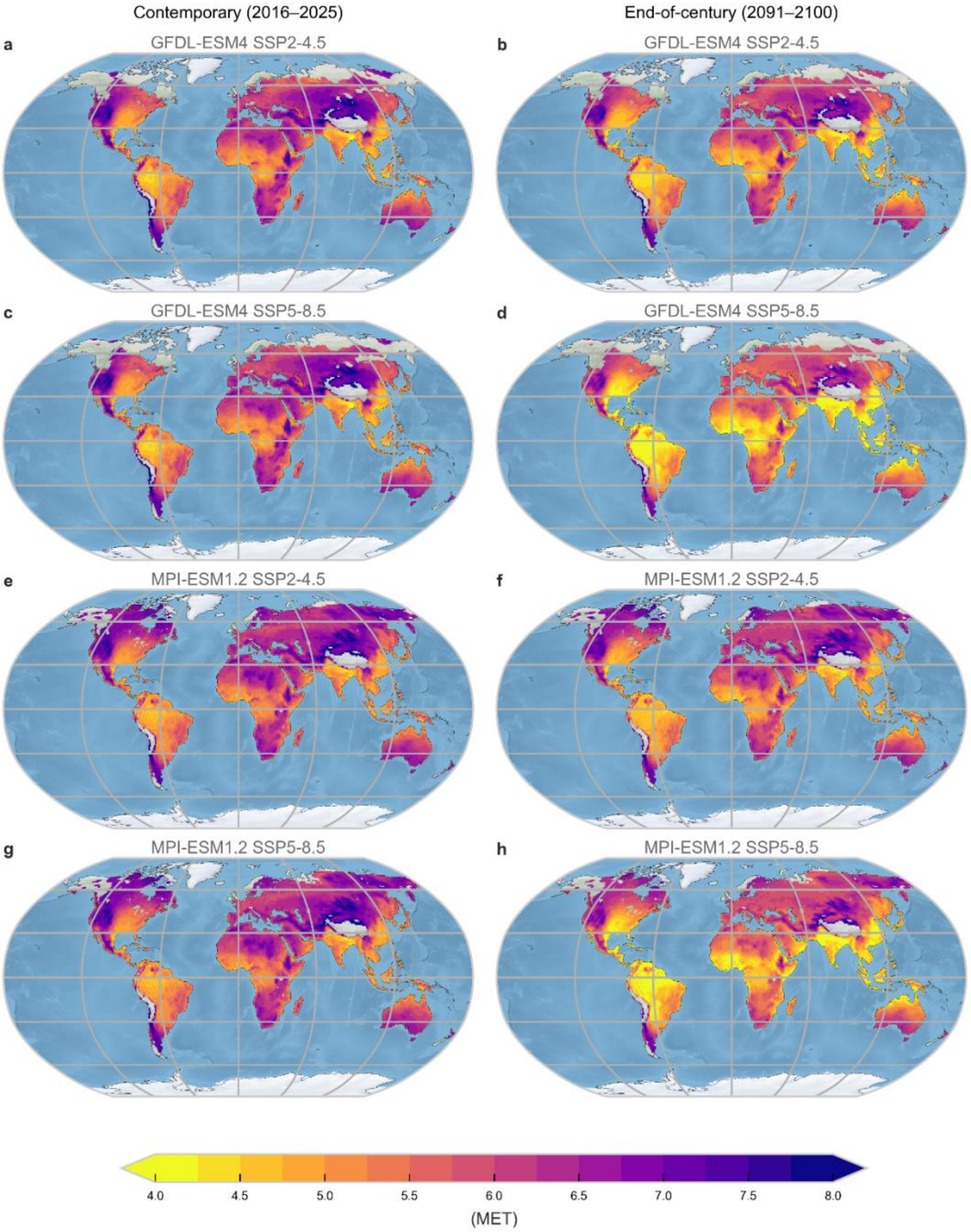

Figure S9: Global maps of median M_{max} for contemporary (2016–2025) (left) and projected end-of-century (2091–2100) (right) for SSP2-4.5 (a, b, e, f) and SSP5-8.5 (c, d, g, h). All analyses are based on warm time (T_{air} >25°C) and using young healthy female adults. 3-hourly CMIP6 data are from GFDL-ESM4 and MPI-ESM1.2 (~1° × 1.25° atmosphere/land grid) following SSP2-4.5 and SSP5-8.5. Areas with no data indicate locations that do not reach T_{air} >25°C in the given decade. Source data for figures are provided with this paper. Made with Natural Earth - free vector and raster map data at naturalearthdata.com.

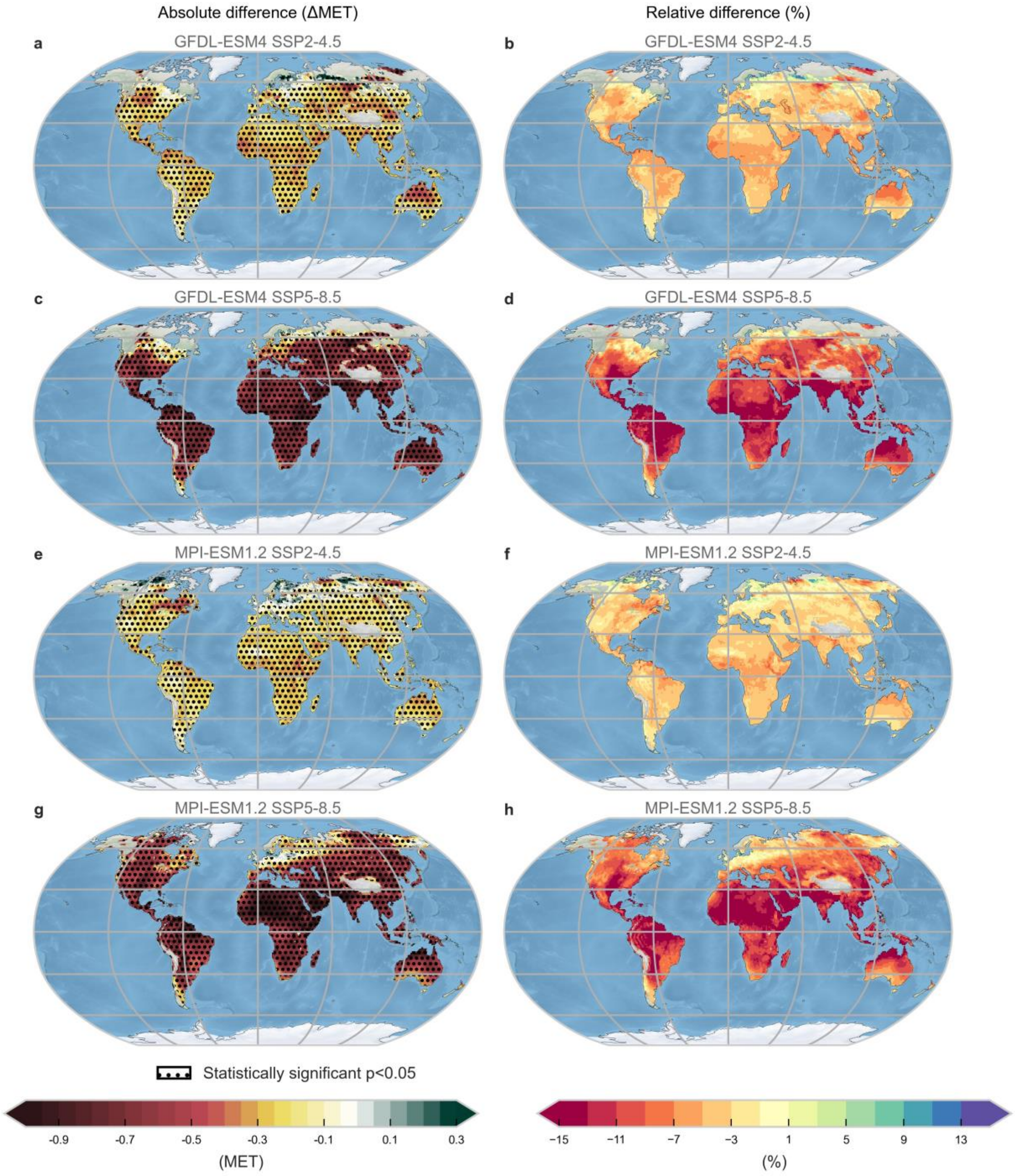

Figure S10: Global maps of absolute (left) and percent (right) differences in median M_{max} between contemporary (2016–2025) and projected data (2091–2100) for SSP2-4.5 (a, b, e, f) and SSP5-8.5 (c, d, g, h). Dotted areas in column 1 (a, c, e, g) indicate where the median difference is statistically significant ($p<0.05$) using two-side Mann-Whitney U test. All analyses are based on warm time ($T_{air}>25°C$) and using young healthy female adult. 3-hourly CMIP6 data are from GFDL-ESM4 and MPI-ESM1.2 (~1° × 1.25° atmosphere/land grid) following SSP2-4.5 and SSP5-8.5. Areas with no data indicate locations that do not reach $T_{air}>25°C$ in the given decade. Significant differences in (a, d) were determined using two-sided Mann-Whitney U tests. Source data for figures are provided with this paper. Made with Natural Earth - free vector and raster map data at naturalearthdata.com.

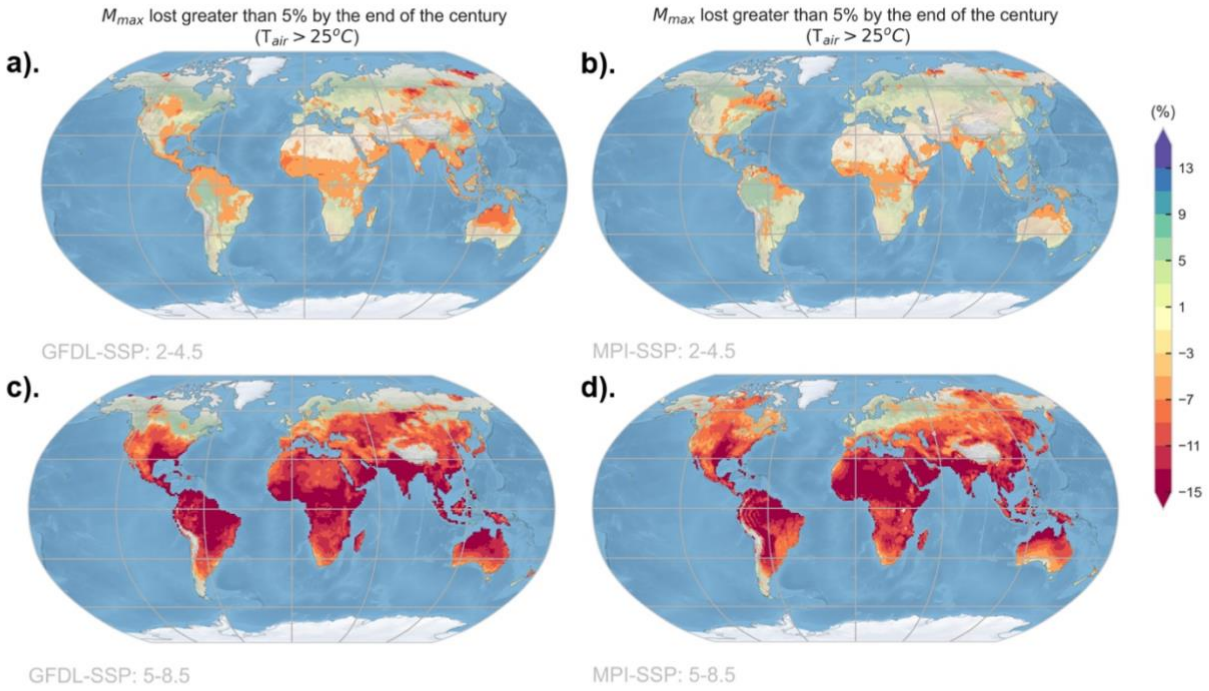

Figure S11: Global map of M_{max} showing areas with %M_{max} loss >5% by the end-of-century (2091–2100) (compared to 2016–2025 baseline). All analyses are based on warm time (T_{air}>25°C) and model young female adults. 3-hourly CMIP6 data are from GFDL-ESM4 (a, c) and MPI-ESM1.2 (b, d) (~1° × 1.25° atmosphere/land grid) following SSP2-4.5 and SSP5-8.5. Source data for figures are provided with this paper. Made with Natural Earth - free vector and raster map data at naturalearthdata.com.

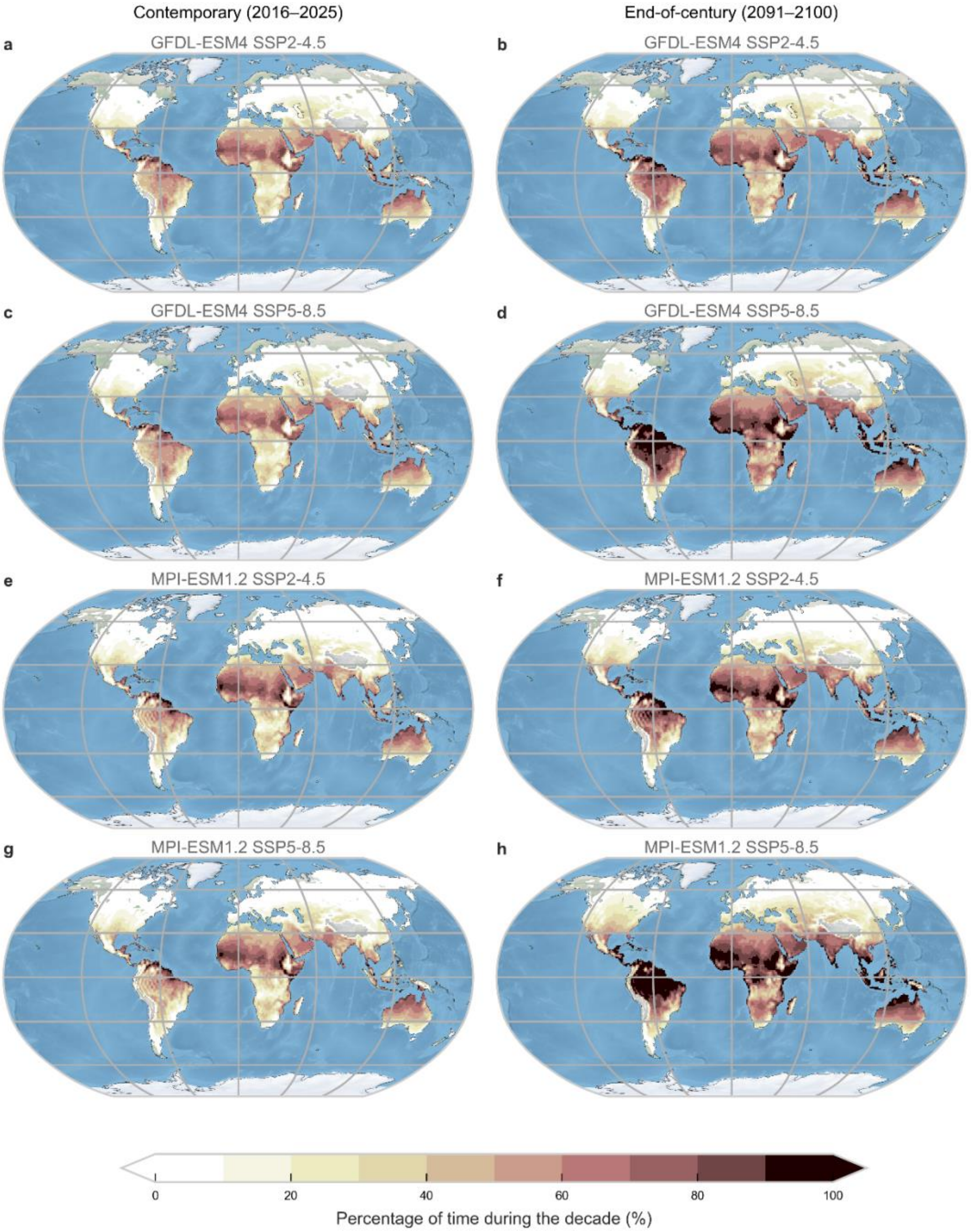

Figure S12: Global maps of percentage of time with T_{air} >25°C for contemporary baseline (2016-2025, left) and (b) end-of-century (2091–2100, right) for SSP2-4.5 (a, b, e, f) and SSP5-8.5 (c, d, g, h). All analyses are based on warm time (T_{air} >25°C) and using young healthy female adult profiles and 3-hourly CMIP6 data from GFDL-ESM4 (b, d) and MPI-ESM1.2 (f, h) (~1º × 1.25º grid) following SSP2-4.5 and SSP5-8.5. Areas with no data indicate locations that do not reach T_{air} >25°C in the given decade. Source data for figures are provided with this paper. Made with Natural Earth - free vector and raster map data at naturalearthdata.com.

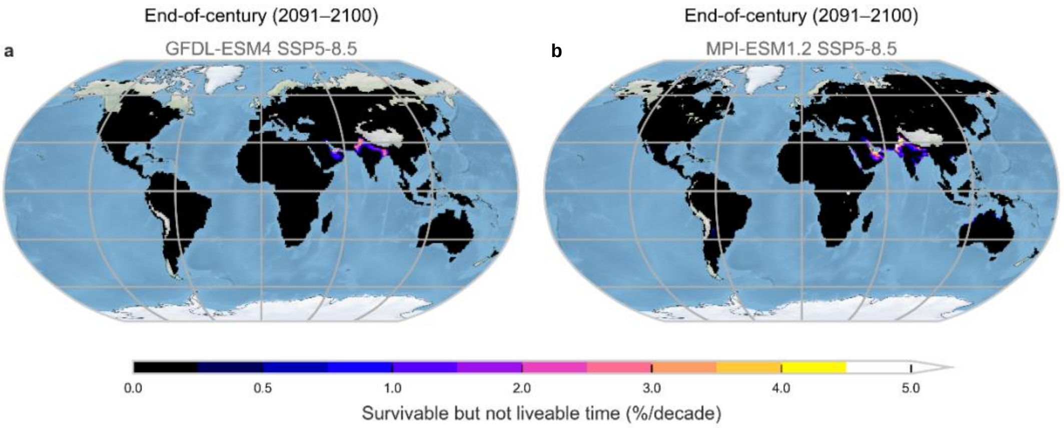

Figure S13: Global map of the percentage of time where conditions are survivable but not liveable for end-of-century (2091–2100). 3-hourly CMIP6 data are from GFDL ESM4 (a) and MPI ESM1.2 (b) (~1° × 1.25° atmosphere/land grid) following SSP5-8.5. Not percent change is found to be survivable but not liveable in SSP2-4.5. Percentages are based on young, healthy female adults. Main changes occur in the Middle East. For example, blue to yellow and white areas show where there is an increase >0.5% in which conditions switch from liveable to only survivable. Source data for figures are provided with this paper. Made with Natural Earth - free vector and raster map data at naturalearthdata.com.

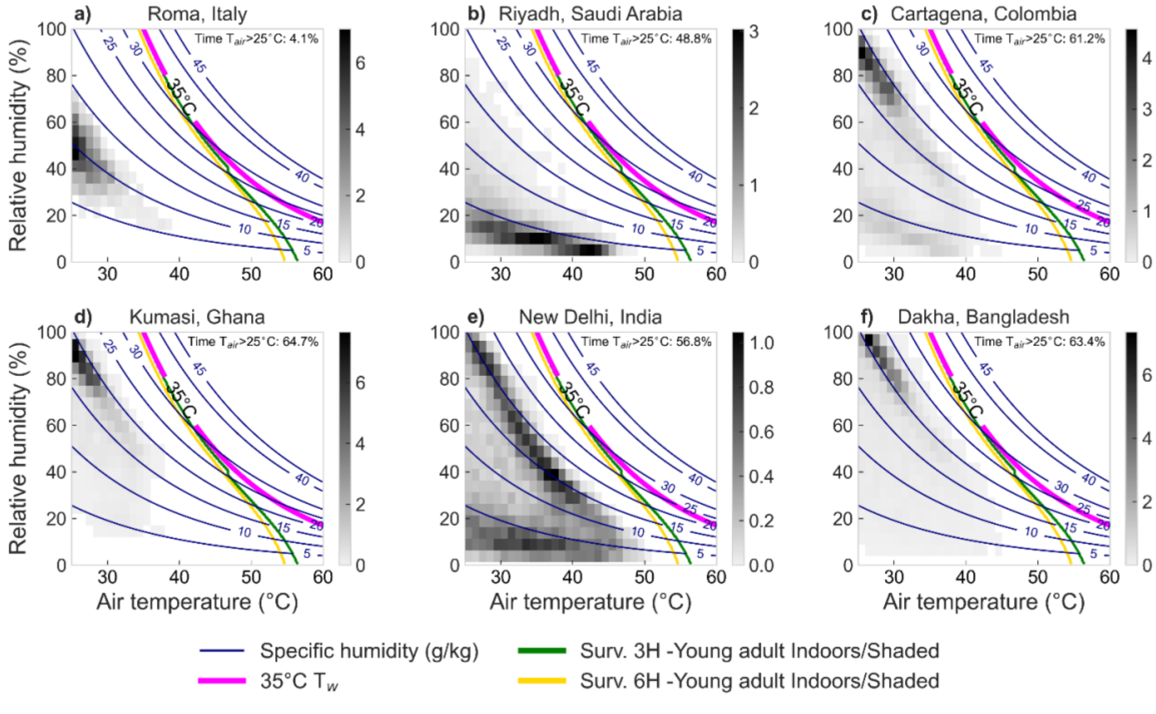

Figure S14: Frequency distribution of 3-hourly T_{air} (x-axis), relative humidity (y-axis), and **specific humidity (dark blue contour lines) for the present decade (2016–2025)** in 6 cities globally with dry (b), medium or mix (a, d, e), and high humid climates (c, d, f). 3-hourly CMIP6 data are from GFDL ESM4 (~1° × 1.25° atmosphere/land grid) following SSP2-4.5. The green and yellow lines indicate the survivability limits for a constant thermal exposure of 3 and 6 hours, respectively. Thick magenta lines indicate T_w=35°C. Source data for figures are provided with this paper.

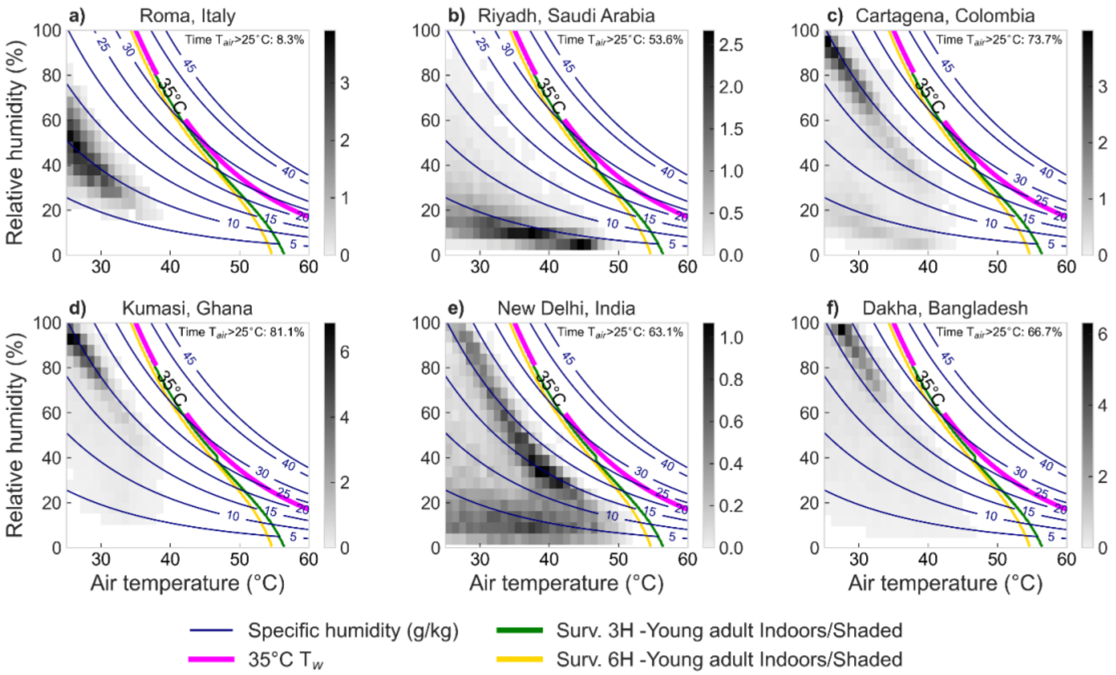

Figure S15: Frequency distribution of 3-hourly T_{air} (x-axis), relative humidity (y-axis), and specific humidity (dark blue contour lines) for the end-of-century decade (2091–2100) in 6 cities globally with dry (b), medium or mix (a, d, e), and high humid climates (c, d, f). Compared to S14, these graphs show that conditions are starting to, and will continue to, approach the T_w=35 ºC line by end-of-century. 3-hourly CMIP6 data are from GFDL ESM4 (~1° × 1.25° atmosphere/land grid) following SSP2-4.5. The green and yellow lines indicate the survivability limits for a constant thermal exposure of 3 and 6 hours, respectively. Thick magenta lines indicate T_w=35°C. Source data for figures are provided with this paper.

Supplementary References:

1. UniData. MetPy: A Meteorological Python Library for Data Analysis and Visualization. *MetPy* Preprint at https://doi.org/10.5065/D6WW7G29.
2. Davies-Jones, R. An efficient and accurate method for computing the wet-bulb temperature along pseudoadiabats. *Mon Weather Rev* **136**, 2764–2785 (2008).
3. Cramer, M. N. & Jay, O. Partitional calorimetry. *J Appl Physiol* **126**, 267–277 (2019).
4. Bouchama, A. *et al.* Classic and exertional heatstroke. *Nat Rev Dis Primers* **8**, 1–23 (2022).
5. Gagge, A. P. & Gonzalez, R. R. Mechanisms of Heat Exchange: Biophysics and Physiology. *Comprehensive Physiology* 45–84 Preprint at https://doi.org/https://doi.org/10.1002/cphy.cp040104 (2011).
6. Havenith, G. & Fiala, D. Thermal Indices and Thermophysiological Modeling for Heat Stress. *Compr Physiol* **6**, 255–302 (2016).
7. Handbook, A. American Society of Heating, Refrigerating, and Air Conditioning Engineers; Atlanta: 1993. Preprint at (1997).
8. Dubois, D. & Dubois, E. F. A formula to estimate the surface area if height and weight are known. *Arch Intern Med* **17**, 863 (1916).
9. Rose, M., Appel, C. & Ritchie, H. Human Height. *Our World in Data; https://ourworldindata.org/human-height* (2013).
10. European Commission, J. R. C. E. Commission. ExpoFacts: the European Exposure Factors Sourcebook. *https://joint-research-centre.ec.europa.eu/expofacts-european-exposure-factors-sourcebook_en* (2013).

11. Phillips, L. J. & Moya, J. Exposure factors resources: contrasting EPA's Exposure Factors Handbook with international sources. *J Expo Sci Environ Epidemiol* **24**, 233–243 (2013).
12. U.S. Environmental Protection Agency. *Exposure Factors Handbook: 2011 Edition*. (2011).
13. Drinkwater, B. L., Bedi, J. F., Loucks, A. B., Roche, S. & Horvath, S. M. Sweating sensitivity and capacity of women in relation to age. *J Appl Physiol Respir Environ Exerc Physiol* **53**, 671–676 (1982).
14. Sherwood, S. C. & Huber, M. An adaptability limit to climate change due to heat stress. *Proceedings of the National Academy of Sciences* **107**, 9552–9555 (2010).
15. Winslow, C.-E. A., Herrington, L. P. & Gagge, A. P. Physiological reactions of the human body to varying environmental temperatures. *American Journal of Physiology-Legacy Content* **120**, 1–22 (1937).
16. Parsons, K. *Human Thermal Environments*. (CRC Press, 2014). doi:10.1201/b16750.
17. ISO, Io. ISO 8996 Ergonomics of Thermal Environment-Determination of Metabolic Heat Production. (2004).
18. McCullough, E. A., Jones, B. W. & Huck, J. A comprehensive database for estimating clothing insulation. *ASHRAE Trans* **91**, 29–47. (1985).
19. Lewis, W. K. The evaporation of a liquid into a gas. *ASME Transactions* **44**, 325–355 (1922).
20. Morris, N. B. *et al.* Electric fan use for cooling during hot weather: a biophysical modelling study. *Lancet Planet Health* **5**, e368–e377 (2021).
21. Foster, J. *et al.* Quantifying the impact of heat on human physical work capacity; part II: the observed interaction of air velocity with temperature, humidity, sweat rate, and clothing is not captured by most heat stress indices. *Int J Biometeorol* **66**, 507–520 (2022).
22. Morris, N. B., English, T., Hospers, L., Capon, A. & Jay, O. The Effects of Electric Fan Use Under Differing Resting Heat Index Conditions: A Clinical Trial. *Ann Intern Med* **171**, 675–677 (2019).
23. Candas, V., Libert, J. P. & Vogt, J. J. Influence of air velocity and heat acclimation on human skin wettedness and sweating efficiency. *J Appl Physiol* **47**, 1194–1200 (1979).
24. Candas, V., Libert, J. P. & Vogt, J. J. Human skin wettedness and evaporative efficiency of sweating. *J Appl Physiol* **46**, 522–528 (1979).
25. Graham, C., Morris, N. B., Harwood, A. E. & Jay, O. Ad libitum water consumption off-sets the thermal and cardiovascular strain exacerbated by dehydration during a 3-h simulated heatwave. *Eur J Appl Physiol* **120**, 391–399 (2020).
26. Périard, J. D., Racinais, S. & Sawka, M. N. Adaptations and mechanisms of human heat acclimation: applications for competitive athletes and sports. *Scand J Med Sci Sports* **25**, 20–38 (2015).
27. Liu, J. *et al.* Heat exposure and cardiovascular health outcomes: a systematic review and meta-analysis. *Lancet Planet Health* **6**, e484–e495 (2022).
28. Ebi, K. L. *et al.* Hot weather and heat extremes: health risks. *The Lancet* **398**, 698–708 (2021).
29. Basile, D. P., Anderson, M. D. & Sutton, T. A. Pathophysiology of acute kidney injury. *Compr Physiol* **2**, 1303 (2012).
30. Ainsworth, B. E. *et al.* 2011 Compendium of Physical Activities: a second update of codes and MET values. *Med Sci Sports Exerc* **43**, 1575–1581 (2011).
31. Wolf, S. T., Cottle, R. M., Vecellio, D. J., Kenney, W. L. & Wolf, S. T. Critical environmental limits for young, healthy adults (PSU HEAT). *J Appl Physiol* **132**, 327–333 (2022).
