# Supplementary material for: A physiological approach for assessing human survivability and liveability to heat in a changing climate
Source: Nat Commun. 2023 Nov 29;14:7653. doi: 10.1038/s41467-023-43121-5 (PMC10687011; doi:10.1038/s41467-023-43121-5)
Supplement: Supplementary file 3 — Reporting Summary [file 41467_2023_43121_MOESM3_ESM.pdf]

nature portfolio

Corresponding author(s): Jennifer Vanos

Last updated by author(s): Oct 17, 2023

Reporting Summary

Nature Portfolio wishes to improve the reproducibility of the work that we publish. This form provides structure for consistency and transparency in reporting. For further information on Nature Portfolio policies, see our Editorial Policies and the Editorial Policy Checklist.

Statistics

For all statistical analyses, confirm that the following items are present in the figure legend, table legend, main text, or Methods section.

n/a	Confirmed	
☒	☐	The exact sample size (*n*) for each experimental group/condition, given as a discrete number and unit of measurement
☒	☐	A statement on whether measurements were taken from distinct samples or whether the same sample was measured repeatedly
☐	☒	The statistical test(s) used AND whether they are one- or two-sided<br>*Only common tests should be described solely by name; describe more complex techniques in the Methods section.*
☒	☐	A description of all covariates tested
☐	☒	A description of any assumptions or corrections, such as tests of normality and adjustment for multiple comparisons
☒	☐	A full description of the statistical parameters including central tendency (e.g. means) or other basic estimates (e.g. regression coefficient) AND variation (e.g. standard deviation) or associated estimates of uncertainty (e.g. confidence intervals)
☐	☒	For null hypothesis testing, the test statistic (e.g. *F*, *t*, *r*) with confidence intervals, effect sizes, degrees of freedom and *P* value noted<br>*Give P values as exact values whenever suitable.*
☒	☐	For Bayesian analysis, information on the choice of priors and Markov chain Monte Carlo settings
☒	☐	For hierarchical and complex designs, identification of the appropriate level for tests and full reporting of outcomes
☒	☐	Estimates of effect sizes (e.g. Cohen's *d*, Pearson's *r*), indicating how they were calculated

Our web collection on statistics for biologists contains articles on many of the points above.

Software and code

Policy information about availability of computer code

Data collection	The models are run across all plausible combinations of air temperature and moisture levels (e.g., RH) for warm conditions, which act as the input data. The only external data comes from CMIP6 data, which were downloaded from https://esgf-node.llnl.gov/search/cmip6/. The input data for the mode for the personal profiles custom-built for this application are provided in the methods, supplemental material, and Zenodo repository.
Data analysis	The model code was developed using Python 3.10.9 and authors thank the teams behind this open-source project, as well as NumPy (v1.23.5), Matplotlib (v3.7), Xarray (v2022.11.0), Pandas (V 1.5.3), Cartopy (V 0.21.1), and MetPy (1.4.1) developers. Custom codes with the model developed for this study and tutorials to reproduce survivability and liveability temperature-humidity matrices are available via Zenodo data repository (https://doi.org/10.5281/zenodo.10020136).

For manuscripts utilizing custom algorithms or software that are central to the research but not yet described in published literature, software must be made available to editors and reviewers. We strongly encourage code deposition in a community repository (e.g. GitHub). See the Nature Portfolio guidelines for submitting code & software for further information.

Data

Policy information about availability of data

All manuscripts must include a data availability statement. This statement should provide the following information, where applicable:

- Accession codes, unique identifiers, or web links for publicly available datasets
- A description of any restrictions on data availability
- For clinical datasets or third party data, please ensure that the statement adheres to our policy

The model output data generated in this study are provided as source data files. Input data included all plausible combinations of air temperature and moisture levels for warm conditions, which we used for generating survivability wet-bulb curves and liveability analyses; therefore, the only external data comes from CMIP6 data. The input data for the mode for the personal profiles custom-built for this application are provided in the methods, supplemental material, and Zenodo repository. The CMIP6 data were downloaded from https://esgf-node.llnl.gov/search/cmip6/. All the source data for figures are provided with this paper as a Source Data file, including the wet bulb temperature matrices from the combinations of air temperature and humidity tested.

Research involving human participants, their data, or biological material

Policy information about studies with human participants or human data. See also policy information about sex, gender (identity/presentation), and sexual orientation and race, ethnicity and racism.

Reporting on sex and gender	NA
Reporting on race, ethnicity, or other socially relevant groupings	NA
Population characteristics	NA
Recruitment	NA
Ethics oversight	NA

Note that full information on the approval of the study protocol must also be provided in the manuscript.

Field-specific reporting

Please select the one below that is the best fit for your research. If you are not sure, read the appropriate sections before making your selection.

☐ Life sciences ☐ Behavioural & social sciences ☒ Ecological, evolutionary & environmental sciences

For a reference copy of the document with all sections, see nature.com/documents/nr-reporting-summary-flat.pdf

Ecological, evolutionary & environmental sciences study design

All studies must disclose on these points even when the disclosure is negative.

Study description	A quantitative experimental modeling study looking at all warm-weather values of wet-bulb temperatures, based on different combinations of temperature and relative humidity (RH), that could lead to conditions that were unsurvivable or unlivable based on a physiological model applied to two different subsets of the population. The human heat balance model was used to determine the limits of survivability and change in ability to perform safe, sustained activity in the heat (liveability). Differences of the new wet bulb survivability limits were compared to a Tw of 35degrees C (differences and range) across all combinations of temperature and RH. Comparisons of mean liveability values completed by contemporary (2016–2016) and future (2091–2100) timescales using two-sided Mann-Whitney tests due to the continuous nature of the variable, yet it not being always normally distributed. We used a significance level of <0.05. Future liveability tests were replicated for two climate models and two RCP-SSP combinations. Shifts in distributions in liveability were compared between current/contemporary and future climates.
Research sample	The two populations represented by the model are young healthy adults of small body size (thus representing more women) and older adults. These are demonstrations of the use of the model applied to climate data. No statistical comparisons we completed between the two groups as the model was ran across all plausible warm weather situations.
Sampling strategy	NA
Data collection	Data were downloaded from https://esgf-node.llnl.gov/search/cmip6/.
Timing and spatial scale	CMIP6 data are for the years 2016–2026 and 2091–2100, with a spatial scale of (~1º × 1.25º atmosphere/land grid)
Data exclusions	No data were excluded in the survivability analysis. We excluded moderate and cooler data from the liveability analysis (<25degrees

Data exclusions	C).
Reproducibility	Code has been made publicly available and has been tested by others not involved in the paper to reproduce the results obtained here. All figures and results can be reproduced through the source files.
Randomization	NA
Blinding	NA

Did the study involve field work? ☐ Yes ☒ No

Reporting for specific materials, systems and methods

We require information from authors about some types of materials, experimental systems and methods used in many studies. Here, indicate whether each material, system or method listed is relevant to your study. If you are not sure if a list item applies to your research, read the appropriate section before selecting a response.

Materials & experimental systems

n/a	Involved in the study
☒	☐ Antibodies
☒	☐ Eukaryotic cell lines
☒	☐ Palaeontology and archaeology
☒	☐ Animals and other organisms
☒	☐ Clinical data
☒	☐ Dual use research of concern
☒	☐ Plants

Methods

n/a	Involved in the study
☒	☐ ChIP-seq
☒	☐ Flow cytometry
☒	☐ MRI-based neuroimaging
